# Surgery for colloid carcinoma of the pancreas with portal vein tumor thrombus: a case report

**DOI:** 10.1186/s40792-020-01025-2

**Published:** 2020-10-01

**Authors:** Daisuke Udagawa, Motohide Shimazu, Tadayuki Sakuragawa, Shotaro Maruyama, Yusuke Uchi, Eisuke Miura, Yohei Masugi, Michiie Sakamoto

**Affiliations:** 1 Department of Surgery, Tama Kyuryo Hospital, 1491 Shimooyamadacho, Machida-shi, Tokyo, 194-0297 Japan; 2 Department of Pathology, Keio University School of Medicine, 35 Shinamomachi, Shinjuku-ku, Tokyo, 160-8582 Japan

**Keywords:** Portal vein tumor thrombus, Pancreatic colloid carcinoma, Pancreatoduodenectomy, Resection of the portal vein

## Abstract

**Background:**

The portal vein is occasionally invaded by advanced malignant tumors in the pancreatic head region. However, pancreatic cancer rarely has portal vein tumor thrombi. We report a case of pancreatic cancer with a massive portal vein tumor thrombus undergoing pancreatoduodenectomy with combined resection of the portal vein.

**Case presentation:**

A 71-year-old man visited a clinic with complaints of abdominal discomfort and vomiting. Gastroscopy showed a massive tumor in the duodenum. He was referred to our hospital for further examinations and treatment. The CT showed a low-density tumor with a maximum diameter of 10 cm located on the pancreas head. A tumor widely invaded the duodenum and had a 6-cm portal vein tumor thrombus. MRCP did not show obvious stenosis of the pancreatic duct due to tumor invasion. There were no findings suggesting distant metastases. Biopsy of the duodenum revealed adenocarcinoma. He was diagnosed with primary pancreatic cancer or duodenal cancer with portal vein tumor thrombus and underwent pancreatoduodectomy with resection and reconstruction of the portal vein. He suffered no postoperative complications and was discharged 2 months after surgery. The final histopathological diagnosis was pancreatic colloid carcinoma. He received adjuvant chemotherapy, but died 16 months after surgery.

**Conclusions:**

Colloid carcinoma of the pancreas is rare, and pancreatic carcinoma seldom forms a portal vein tumor thrombus. We experienced a very rare case of pancreatic colloid carcinoma with portal vein tumor thrombus and performed radical resection of the pancreas and portal vein.

## Background

The portal vein occasionally becomes invaded by advanced malignant tumors in the pancreatic head region. However, pancreatic cancer rarely has a portal vein tumor thrombus [1]. We report a case of pancreatic colloid carcinoma with a portal vein tumor thrombus with some relevant literature review.

## Case presentation

**Fig. 1:**
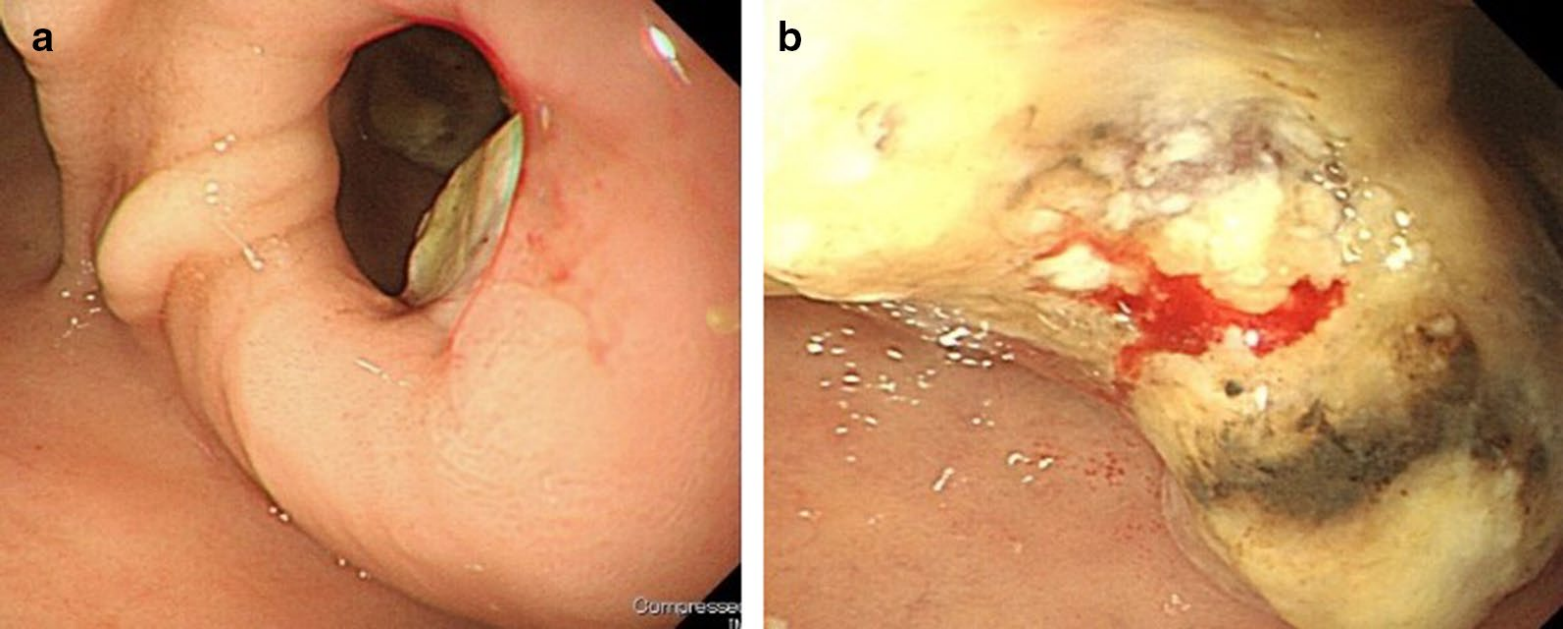
Upper gastrointestinal endoscopy. **a** Pylorus observed from the stomach side. **b** Duodenal bulb lumen. An irregular tumor occupying the lumen of the duodenal bulb was revealed. The biopsy result was adenocarcinoma

**Fig. 2:**
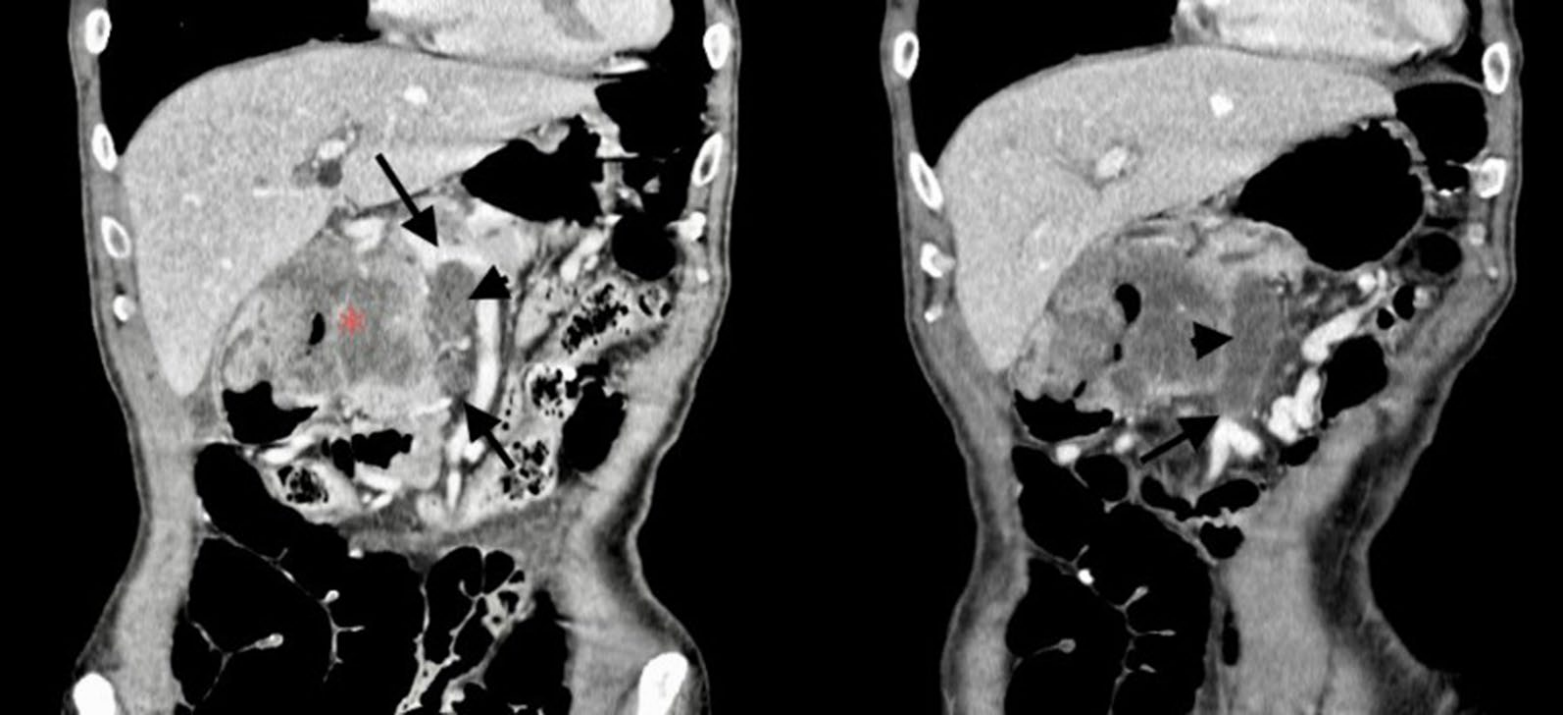
Abdominal enhanced CT. Approximately 10 cm tumor with poor contrast effect was observed in the pancreatic head area (*). A tumor thrombus (arrowhead) of about 6 cm was observed from the superior mesenteric vein (up arrow) to the junction of the portal splenic vein (down arrow)

**Fig. 3:**
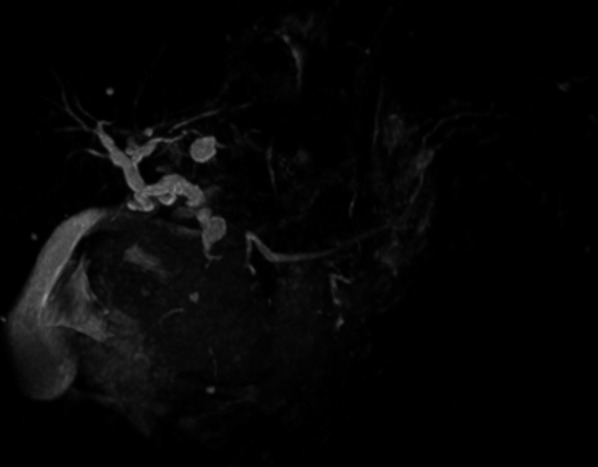
Abdominal MRCP. A large tumor was observed in the head of the pancreas, but no significant dilation of the common bile duct and the main pancreatic duct was observed

**Fig. 4:**
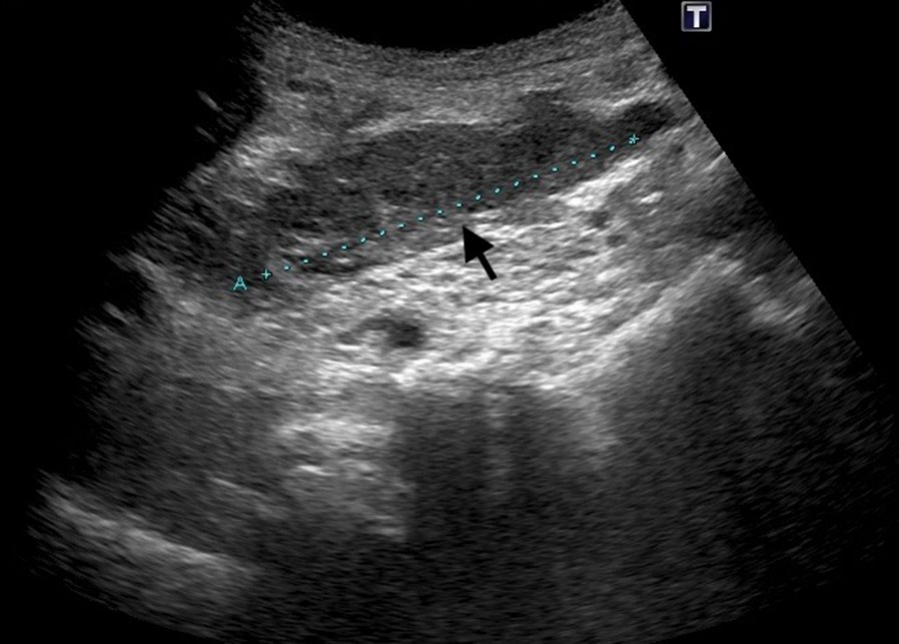
Abdominal ultrasonography. There was an echogenic region in the lumen of the blood vessel at a section less than 6 cm from the upper end of the superior mesenteric vein, which was considered a tumor thrombus (arrow)

The patient is a 71-year-old man who presented with abdominal discomfort and vomiting after a meal. Upper gastrointestinal endoscopy revealed a tumor in the duodenal bulb, and he was referred to our hospital for further examination and treatment. Laboratory tests revealed anemia, elevation of CEA (87.0 ng/ml) and CA19-9 (136 ng/ml), and a normal total bilirubin value (0.7 mg/dl). He underwent upper gastrointestinal endoscopy again, which revealed an irregular tumor occupying the lumen of the duodenal bulb, and the 2nd portion could not be observed. The biopsy result was adenocarcinoma (Fig. 1a, b). CT scan revealed a 10-cm tumor with poor contrast effect in the pancreatic head region, and a 6-cm tumor thrombus was observed from the superior mesenteric vein to the junction of the portal and splenic vein (Fig. 2a, b). Invasion into the gastroduodenal artery was suspected, but no invasion into the celiac or superior mesenteric arteries was revealed. MRCP showed no significant dilation of the common bile duct or main pancreatic duct (Fig. 3). In abdominal ultrasonography, the lesion invading the superior mesenteric vein was considered to be a tumor thrombus with an echogenic region in the vascular lumen (Fig. 4). After admission, frequent blood transfusions were required due to bleeding from the tumor. There were no problems with cardiopulmonary function and no evidence of distant metastasis. He was diagnosed with primary pancreatic cancer or primary duodenal cancer with portal vein tumor thrombus. We planned to perform pancreatoduodenectomy combined with portal vein resection to cure and relieve the symptoms.

**Fig. 5:**
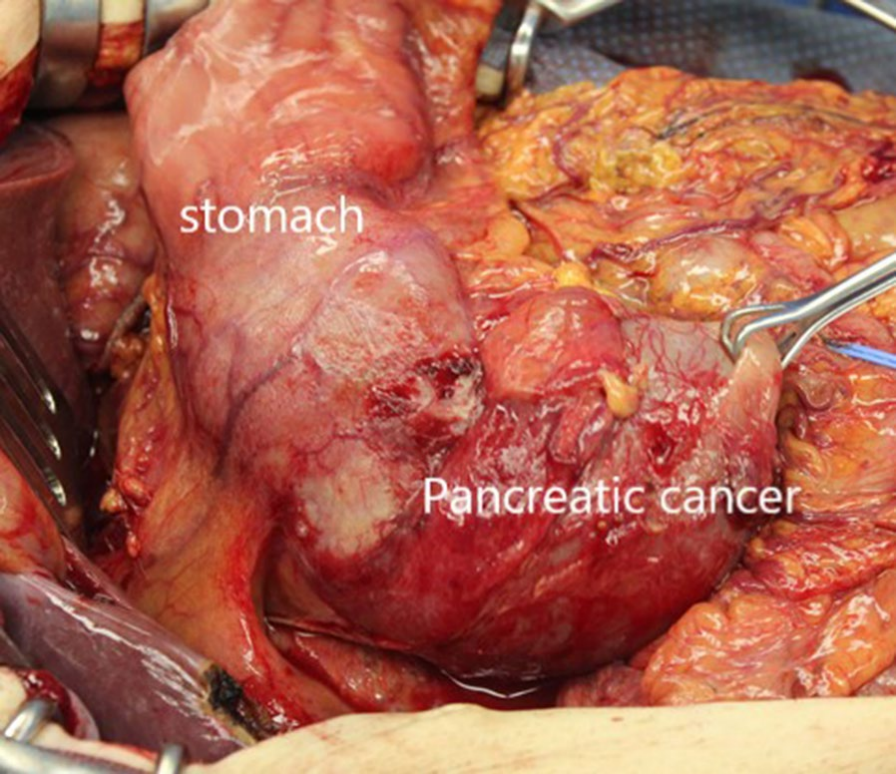
Operation findings. 1. A huge tumor was found in the pancreatic head

**Fig. 6:**
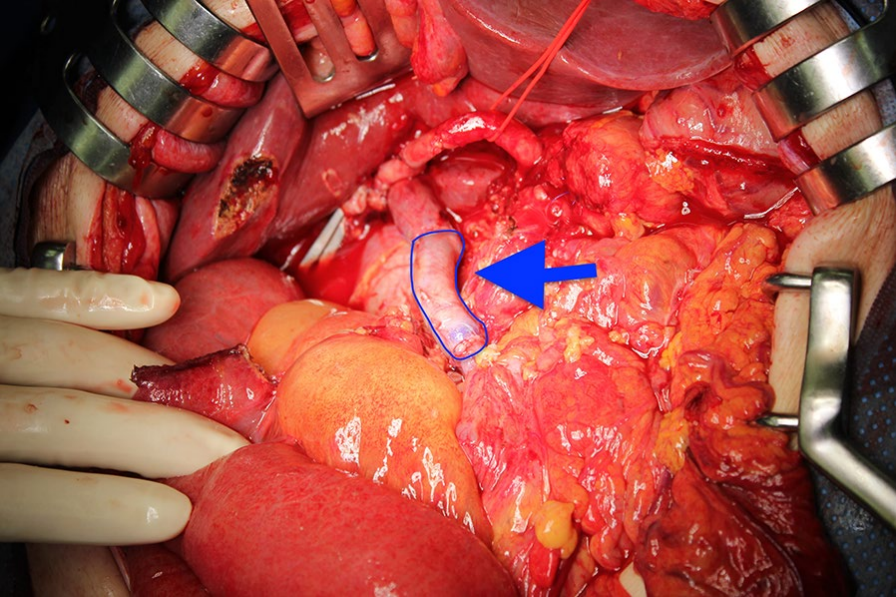
Operation findings 2. After specimen removal, portal vein reconstruction was performed using a right external iliac vein graft (arrow)

**Fig. 7:**
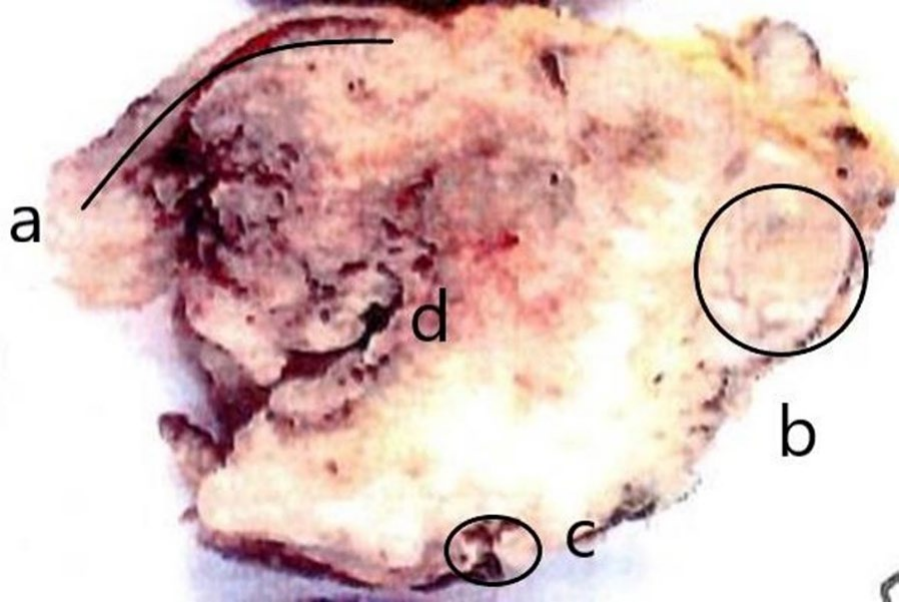
Section of lesion. A tumor occupying the head of the pancreas and invading the duodenal lumen. **a** Mucosa of duodenum, **b** portal vein, **c** common bile duct, **d** tumor

**Fig. 8:**
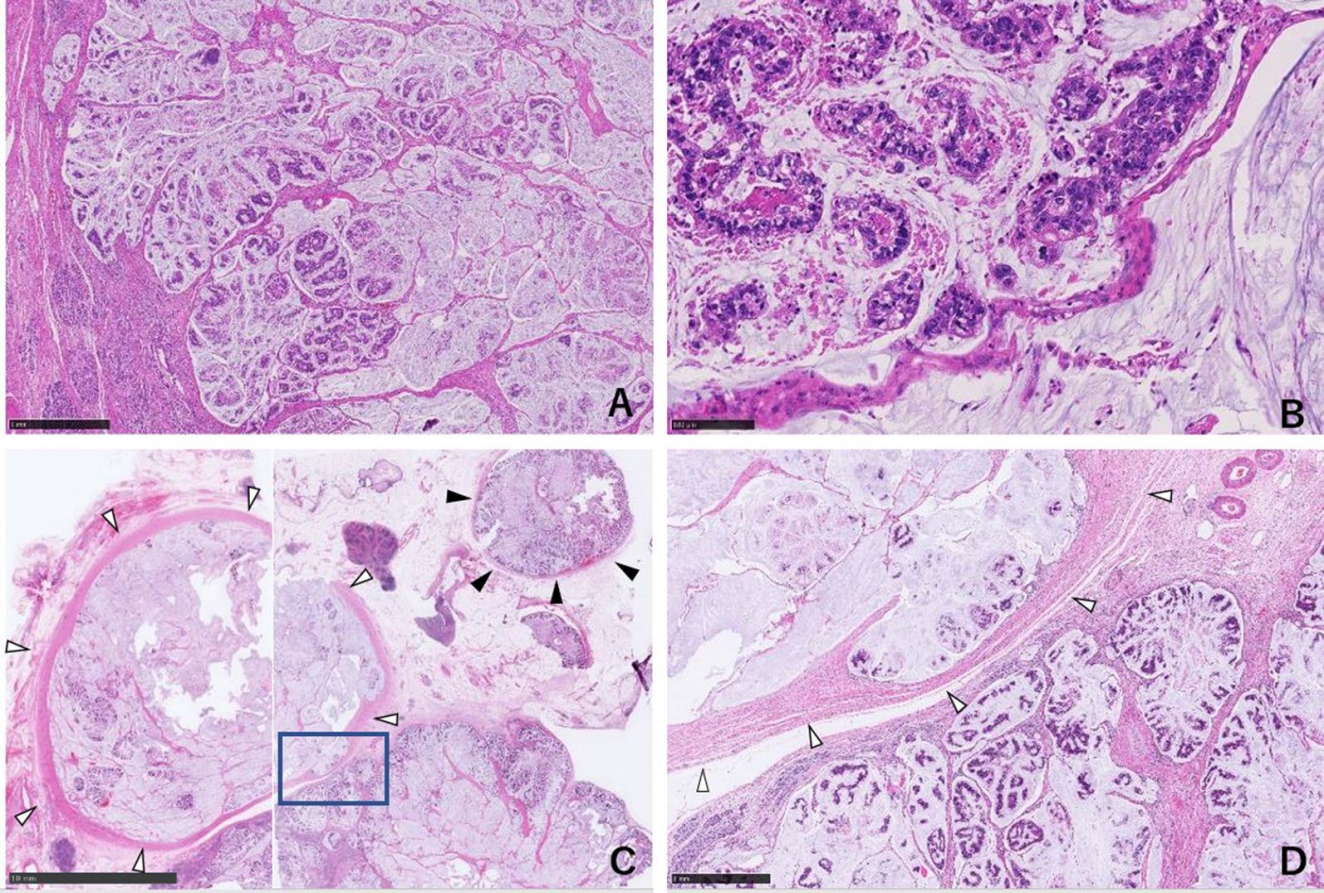
Pathological findings. **a** Medium magnification image: abundant mucus and cell mass floating in the mucus lake are recognized. **b** High-magnification image: cells with densely stained and swollen irregular nuclei form clumps and float with some connectivity. This is a finding of colloid cancer. **c** Portal vein: the portal vein (white arrowheads) was full of tumor, and the small vessel around the tumor (black arrowheads) was also full of tumor. **d** The high-magnification image of **c** (rectangle). There was no distinct evidence of the tumor directly involving into the portal vein

At operation, a large tumor was found in the head of the pancreas and the descending duodenum (Fig. 5). GDA was not involved. There were no obvious findings of peritoneal dissemination or liver metastasis, so we performed pancreaticoduodenectomy, with combined resection of the portal vein replaced by the right external iliac vein graft (Fig. 6). The caudal limit of the tumor thrombus was just above the first branch of the SMV, and the SMV was cut at one orifice. The cranial side of the portal vein was cut in the hepatoduodenal ligament, and the splenic vein was ligated. The portal vein was reconstructed by the interposition of the right external iliac vein graft. The portal vein flow was gradually interrupted by thrombosis formation so that collateral vessels developed. SMV flow was completely blocked, and the flow seemed to run through collateral vessels. This made surgery difficult due to hemorrhage. On the other hand, temporary bypass was not necessary because collateral vessels were well developed. The operation time was 615 min, and blood loss was 2405 g. The resected specimen showed a large tumor occupying the head of the pancreas and invading the duodenal lumen (Fig. 7). The tumor showed a gelatinous appearance on cross section and was mainly located in the pancreas head. The tumor size was 105 × 70 × 100 mm in three dimensions. Histopathological findings showed colloid carcinoma of the pancreas, as adenocarcinoma cells floating in the mucus lake were observed (Fig. 8a, b). No adenomatous lesions were observed in the adjacent duodenum mucosa. There was a tumor thrombus in the portal vein. Tumor thrombi were also observed in small vessels within and around the tumor bed (Fig. 8c). Because we did not observe any distinct evidence of direct cancer involvement into the portal vein (Fig. 8d), we considered that this tumor thrombus in the portal vein was formed by intravascular extension through venous vessels. According to the general rules of the AICC/UICC 8th edition TNM staging system, the tumor was described as pT3N0M0. The resection margin was positive for the tumor thrombus at the proximal stump of the portal vein. Immunohistochemistry studies showed that it was CK7+, CK20−, CA19-9+, CK19+, and CDX-2+, which were consistent with colloid carcinoma of the pancreas.

The postoperative course was uneventful, and there were no complications, including pancreatic fistula. He was discharged from the hospital approximately 2 months after the operation after moving to the rehabilitation ward. He moved to the rehabilitation ward not only for medical reasons, but also for social reasons. After 6 weeks of surgery, S-1 was administered as adjuvant chemotherapy, which was discontinued and switched to gemcitabine monotherapy as he had severe diarrhea. Liver metastases appeared 12 months postoperatively, so gemcitabine was used in combination with nab-paclitaxel for 2 months. However, his physical status worsened, and chemotherapy was discontinued. He died at home 16 months after the operation.

## Discussion

Since the portal vein and superior mesenteric artery run close to the head of the pancreas, malignant tumors arising in this area occasionally invade these important blood vessels. Primary pancreatic cancer rarely has portal vein tumor thrombus. A search for “pancreatic carcinoma” and “portal vein tumor thrombus” in the Central Medical Journal and PubMed was conducted, and 16 cases of pancreatic cancer with a portal vein tumor thrombus were reported, of which only three cases were primary pancreatic ductal carcinomas [1, 2], all of which were resected. This is the 4th case of pancreatic ductal carcinoma with a portal vein tumor thrombus.

Table 1 summarizes the cases of pancreatic ductal carcinoma with portal vein tumor thrombus, including the current case. Compared with the other three cases, this case had a larger tumor diameter and longer tumor thrombus length. In all cases, resection combined with portal vein was performed, but vein graft interposition was performed only in this case. The likelihood of recurrence pattern, timing of recurrence of pancreatic cancer with portal vein tumor thrombosis has not been reported. There is no general guideline for resection for portal vein tumor thrombus. However, there are some reports that if it can be resected according to pancreatic cancer with portal vein invasion, surgery can be performed without any problem [1, 2]. In this case, gastrointestinal bleeding was caused by the tumor so that resection was also necessary as an oncologic emergency.

**Table 1:** Cases of pancreatic ductal carcinoma with portal vein tumor thrombus

	Author	Year	Tumor size (cm)	Thrombus size (mm)	Operation	Pathology	Prognosis	Recurrent site	Adjuvant chemo
1	Yamato	2009	3.5 × 3.5	15	DP + PVR	Por	4 months dead	Local	None
2	Yamato	2009	2.9 × 1.9	35	SSPPD + PVR	Tub2	19 months arrive	Liver	GEM → S-1
3	Murase	2015	2.7 × 1.6	22	SSPPD + PVR	Tub1	27 months dead	Local Lung	S-1
4	Udagawa	2020	10.5 × 7.0	60	PD + PVR	Muc (colloid)	16 months dead	Liver	S-1 → GEM → GEM + nabPTX

There have been more reports on pancreatic tumors other than ductal carcinoma. According to Yamato et al. [2] 5 cases of endocrine carcinoma [2–6], 3 cases of acinar cell carcinoma [7–9], 2 cases of solid pseudopapillary tumor [10, 11], 2 cases of IPMC [12], 1 case of pancreatoblastoma [13] and 1 case melanoma [14] have been reported. In the case of melanoma and endocrine carcinoma, the survival period was 25 months on average, but in the other cases, no case showed long-term survival, of which the longest survival was only 20 months.

The present case was a pancreatic colloid carcinoma, which is rare among pancreatic ductal carcinomas [15, 16]. The frequency of pancreatic colloid carcinoma was reported as 0.6% of all pancreatic cancer and 1.4% of invasive pancreatic ductal carcinoma [17]. In a pathological study of 24 cases of colloid carcinoma of the pancreas, Seidel et al. reported that all cases were derived from IPMN [18].

According to Adsay et al. 9 cases of 17 colloid carcinomas were derived from IPMN [19], indicating that there are many IPMN-derived pancreatic mucinous carcinomas. In this case, no IPMN component was observed in either the invasive or noninvasive parts of the tumor, and it can be diagnosed as de novo colloid carcinoma in the invasive ductal carcinoma portion. On the other hand, the biological and clinical features of IPMN-derived colloid carcinoma and de novo colloid carcinoma have not been clarified. Nakahashi [20] et al. summarized 23 cases of de novo colloid carcinoma of the pancreas. They showed that tumors were often located on the pancreas head, and the average tumor size was 51.1 mm. According to Sakoda [16], metastasis often occurs in the liver; however, they also reported a case of lung metastasis. Adsay [19] et al. reported that the 5-year survival rate of colloid carcinoma was 57%, which is better than that of invasive ductal carcinoma. However, their data may include cases of IPMN-derived colloid carcinoma. The accumulation of cases of colloid carcinoma as pancreatic ductal carcinoma is still limited, and the relationship between portal vein tumor thrombus and pancreatic colloid cancer is unclear. To our knowledge, there has been no report of a case of pancreatic colloid carcinoma complicated with portal vein tumor thrombus.

Hepatic metastasis seems to easily occur via hematogenous transfer in pancreatic cancer with portal vein tumor thrombus. The incidence of liver metastasis has not been reported in cases with portal vein tumor thrombosis, so it is not clear whether liver metastases after surgery occur more frequently in cases with portal thrombus than in cases with portal invasion. In this case, chemotherapy administration was started with S-1 according to the recommended postoperative chemotherapy for pancreatic cancer in the guidelines of the Japan Pancreas Society. However, in the early postoperative period, liver metastases were noted. If the general condition allows, it may be necessary to start with stronger chemotherapy, such as FOLFIRINOX, even though effective therapy after surgery for pancreatic cancer with portal vein tumor thrombosis has not been reported.

## Conclusions

We report a very rare case of pancreatic colloid carcinoma with a portal vein tumor thrombus. To our knowledge, there is no report of such a case.

## Abbreviations

CEA: Carcinoembryonic antigen
CA19-9: Carbohydrate antigen 19-9
PM: Proximal margin
DM: Distal margin
RM: Radial margin
MRCP: Magnetic resonance cholangiopancreatography
IMPC: Intraductal papillary mucinous carcinoma
IPMN: Intraductal papillary mucinous neoplasm
DP: Distal pancreatectomy
PVR: Portal vein resection
SSPPD: Subtotal stomach-preserving pancreaticoduodectomy
PD: Pancreaticoduodectomy
S-1: Tegafur/gimeracil/oteracil
GEM: Gemcitabine
nabPTX: Nab-paclitaxel
